# PPARs in Alveolar Macrophage Biology

**DOI:** 10.1155/2007/23812

**Published:** 2007-09-04

**Authors:** Monica R. Smith, Theodore J. Standiford, Raju C. Reddy

**Affiliations:** Division of Pulmonary and Critical Care Medicine, Department of Internal Medicine, University of Michigan Medical Center, Ann Arbor, MI 48109-2200, USA

## Abstract

PPARs, most notably PPAR-*γ*, play a crucial role in regulating the activation of alveolar macrophages, which in turn occupy a pivotal place in the immune response to pathogens and particulates drawn in with inspired air. In this review, we describe the dual role of the alveolar macrophage as both a first-line defender through its phagocytotic activity and a regulator of the immune response. Depending on its state of activation, the alveolar macrophage may either enhance or suppress different aspects of immune function in the lung. We then review the role of PPAR-*γ* and its ligands in deactivating alveolar macrophages—thus limiting the inflammatory response that, if unchecked, could threaten the essential respiratory function of the alveolus—while upregulating the cell's phagocytotic activity. Finally, we examine the role that inadequate or inappropriate PPAR-*γ* responses play in specific lung diseases.

## 1. INTRODUCTION

Peroxisome proliferator-activated receptors (PPARs) are members of the nuclear-receptor superfamily. Their name derives from the first-discovered member of the class, PPAR-*α*, whose activation induces proliferation of peroxisomes in the liver; no similar effect is seen with other members of the class, however. These receptors act as transcription factors, forming heterodimers with the retinoid X receptor and then binding to specific response elements (PPREs) in the promoter regions of the genes they regulate. When activated by appropriate ligands, PPARs undergo a conformational change that leads to release of corepressors and binding of coactivator molecules, with consequent increases in transcription of the genes involved. Some evidence suggests that in the absence of activating ligands, PPARs may bind corepressors and downregulate expression of genes with PPRE-containing promoters [1].

There are three PPAR isoforms: PPAR-*α*, PPAR-*γ*, and PPAR-*β*/*δ*. Each of the isoforms is the product of a different gene. PPAR-*β*/*δ* is expressed in almost every tissue of the body. PPAR-*α* is most commonly thought of in connection with hepatocytes and PPAR-*γ* with adipocytes, but in fact both are expressed in a variety of cells and tissues. Broadly speaking, PPAR-*α* regulates lipid metabolism, lipoprotein formation and transport as well as lipid catabolism, whereas PPAR-*β*/*δ* promotes lipid oxidation, and PPAR-*γ* promotes adipogenesis [2]. Each has other functions in specific tissues, however. For example, PPAR-*α* inhibits proliferation of vascular smooth muscle cells in response to injury [3] and antagonizes the effects of angiotensin II on the vascular wall [4]. In skin, PPAR-*β*/*δ* induces terminal differentiation of keratinocytes [5]. Activation of PPAR-*γ* in endothelial and vascular smooth muscle cells likewise inhibits expression of the angiotensin II receptor [6] and lowers blood pressure in hypertensive mice [7]. PPAR-*γ* agonists have also been shown to exert antiproliferative effects on a variety of cancer cells [8]. This has led to widespread discussion of their possible usefulness in cancer therapy (e.g., for breast cancer [9]) and even to a few early clinical trials.

All three PPARs have significant anti-inflammatory roles in cells of the immune system. PPAR-*γ* has been found in monocytes/macrophages [10, 11], neutrophils [12], dendritic cells [13], B [14] and T [15–17] lymphocytes, eosinophils [18], natural killer cells [19], and mast cells [20]. PPAR-*γ* downregulates expression of a number of proinflammatory mediators while upregulating expression of anti-inflammatory mediators (reviewed in [21]). PPAR-*α* is found in essentially all the same cells as PPAR-*γ* with the notable exception of (mature) dendritic cells and alveolar macrophages [22]. Among the many anti-inflammatory effects of PPAR-*α* that have been demonstrated is inhibition of airway inflammation induced by lipopolysaccharide [23] and of the inflammatory ear-swelling response to leukotriene B_4_ [24]. Furthermore, the acute anti-inflammatory effects of the anticholesterolemic drug simvastatin have been shown to be mediated by PPAR-*α* [25]. The role of PPAR-*β*/*δ* in the immune system has been less extensively investigated and while alveolar macrophages have been found to express PPAR-*β*/*δ* [22], no studies to our knowledge have demonstrated the functional importance of this receptor in these cells. PPAR-*β*/*δ* has been shown, however, to decrease the expression of proinflammatory mediators by other types of macrophages [26].

Monocytes are produced in the bone marrow but, under the influence of chemoattractant molecules, migrate to various tissues of the body where they differentiate into macrophages and other cells of the immune system. The amount of PPAR-*γ* in monocytes is relatively low [27] but increases sharply during differentiation [28]. Furthermore, PPAR-*γ* agonists stimulate monocyte-macrophage differentiation [27, 29]. The resulting macrophages play key roles in regulation of the immune process. Macrophages are best recognized as phagocytes, but their secretion of either anti-inflammatory or proinflammatory mediators, depending on their own state of activation, plays a crucial role in regulation of immune system activity. Phenotypic characteristics of macrophages differ depending upon the tissue in which they differentiate and remain. This is particularly true with macrophages of the alveolus which differ substantially from peritoneal macrophages or macrophages differentiated in vitro from blood-borne monocytes.

In this review, we examine the role of PPARs, focusing predominantly on the PPAR-*γ* subtype, in regulating the activities of alveolar macrophages, which occupy a pivotal spot both as primary phagocyte and as primary regulator of the immune system's response to pathogens and particulates that reach the alveolus through inspired air. We also examine ways in which inadequate or inappropriate PPAR-*γ* responses can contribute to diseases of the lung.

## 2. THE ALVEOLAR MACROPHAGE: PIVOTAL REGULATOR OF IMMUNE RESPONSE

The lung is constantly exposed to noxious agents, both living pathogens and nonliving particulates, that are drawn into the alveolus with inspired air. The alveolar macrophage represents the first line of defense against these agents. Yet the gas-exchange function of the alveolus depends crucially on the thinness and integrity of the structures separating the air space from the pulmonary capillary. An inflammatory response, with edema and perhaps subsequent fibrosis, would severely impact this essential function. Thus, while inflammation may at times be necessary to eradicate invading pathogens, this response must be strictly regulated, as an inflammatory response to every arriving particle or organism would substantially diminish the lung's functional capacity.

### 2.1. Alveolar macrophages: origin and function

There are two major types of resident immune cells in the alveolus: dendritic cells and macrophages. Neutrophils, eosinophils, lymphocytes, and natural killer cells are also present but tend to be less prominent in the absence of overt inflammation. The dendritic cell, which forms part of the alveolar lining, mediates adaptive immunity. Indeed, it is the dendritic cell that presents antigens to other effector cells of the adaptive immune system and thus induces an antigen-specific response. The macrophage is the primary mediator of the innate immune response that does not require recognition of a specific antigen.

Most alveolar macrophages are derived from circulating monocytes. These monocytes are recruited into the lung, where they differentiate into macrophages under influence of the lung environment. In patients who have received bone marrow transplants, macrophages with the donor genotype replace those with host genotype; kinetics indicate an average macrophage lifespan of 81 days [30]. There is also evidence, however, for proliferation of differentiated macrophages within the alveolus, since replicating macrophages can be observed in bronchoalveolar lavage fluid and are more common in smokers and others with chronic lung inflammation [31]. Observations during acute lung inflammation induced by heat-killed Bacillus Calmette-Guérin indicate that even though local proliferation increased approximately 3-fold, the influx of monocytes was eight times as great [32]. In the normal steady state, greater than 70% of the macrophages are derived from circulating monocytes [33]. Substances known to induce the monocyte-macrophage transition include 1,25-dihydroxycholecalciferol and IL-10, as well as serum factors that remain less well defined [34–37].

The most obvious role of the alveolar macrophage is as a phagocyte. Phagocytes engulf viruses, bacteria, fungal cells, and a variety of appropriately sized nonliving particulates. Once engulfed, these particulates may be degraded if they are susceptible to the enzymes of the lysosomal system, as many (but not all) bacteria and fungi are. Otherwise, the particles will remain encapsulated within the macrophages until the latter either die (probably being engulfed by other macrophages), are transferred to lymph nodes draining the site, or are cleared from the airway by the mucociliary system [38].

Phagocytosis of many pathogens is mediated by the macrophage's Toll-like receptors (named for their sequence similarity to the Toll protein that governs dorsal-ventral patterning in *Drosophila* larvae). As recently reviewed by Akira [39], there are multiple Toll-like receptors; each recognizes a different microbial component or pathogen-associated molecular pattern (PAMP) which initiates signaling pathways through selective utilization of intracellular adaptor molecules. Phagocytosis may also be triggered by receptors for complement and the Fc portion of antibodies, thus targeting pathogens that have been recognized by the adaptive immune system [40]. There are also scavenger receptors that facilitate phagocytosis of particles coated with surfactant proteins A and D, which bind to a wide variety of bacteria and opsonize them (i.e., “tag” them for phagocytosis) [41–43]. Finally, there are scavenger receptors that target inhaled particulates that have not otherwise been “tagged” by the immune system or surfactant proteins [44].

Alveolar macrophages are also involved in maintenance and remodeling of lung tissue, on the one hand secreting growth factors and cytokines that stimulate fibroblast proliferation and matrix synthesis and on the other hand producing matrix-degrading proteinases. Macrophage-secreted factors supporting matrix production include transforming growth factor-*β* (TGF-*β*) [45] and insulin-like growth factor-1 [46]. In addition to stimulating fibroblast proliferation, these cytokines stimulate production of collagen and of tissue inhibitors of matrix metalloproteinases (MMPs) while inhibiting metalloproteinase synthesis. A major matrix-degrading enzyme produced by alveolar macrophages is MMP-1 [47], although other MMPs as well as serine and cysteine proteinases also originate in macrophages.

A crucial nonimmune activity of macrophages is maintenance of pulmonary surfactant homeostasis. Surfactant, which serves to prevent alveolar collapse by reducing surface tension, is a mixture of proteins and lipids (mostly phospholipids) secreted by the epithelial cells of the lung [48]. Newly produced and biologically active surfactant takes the form of relatively large protein-lipid aggregates. Over time, however, the mechanical stresses associated with alveolar motion reduce the aggregates' size until they no longer provide effective surface tension reduction. These small, nonactive aggregates are taken up by both the epithelial cell and the alveolar macrophage [49]; most of those taken up by the epithelial cell are recycled, while those taken up by the macrophage are degraded and eliminated [50]. Hence, the macrophage plays a major role in elimination of excess surfactant.

### 2.2. Key role of alveolar macrophages in lung immune system regulation

Factors expressed by the innate immune system, including antibacterial proteins found in the pulmonary surfactant, are relatively noninjurious to the pulmonary epithelium. Only the generation of bactericidal reactive oxygen species is likely to have toxic effects. The adaptive immune system, on the other hand, relies heavily on inflammatory reactions to fight invading pathogens. Thus, in the lung it is desirable to rely on the innate immune system whenever possible.

When macrophages are stimulated by lipopolysaccharide and other microbial components, these cells respond by elaborating substances that upregulate the innate immune system, including chemoattractant molecules that recruit neutrophils and monocytes. Major chemoattractants produced by alveolar macrophages include leukotriene B_4_ [51] and chemokines, particularly CXCL8 (IL-8) and CCL3 [52–54].

Conversely, in most circumstances the alveolar macrophage suppresses adaptive immunity, both through direct actions on the T cell and by inhibiting antigen presentation by dendritic cells. Depletion of alveolar macrophages in mice and rats, followed by antigen challenge, results in a marked increase in production of all antibody classes and in the number of T cells found in the lung and regional lymph nodes [55]. Antigen presenting activity of the dendritic cells is also increased [56]. Macrophages suppress lymphocyte activation via the production of nitric oxide, prostaglandin E_2_, and immunosuppressive cytokines including TGF-*β* and IL-10 [57, 58]. More recently it has been found that these immunosuppressive cytokines are the product of an “alternatively activated,” or “M2,” macrophage induced by the T_H_2 cytokines IL-4 and IL-13 [59, 60].

There will be times when the innate system is overwhelmed and the adaptive system must be activated. A recent elegant paper by Takabayshi et al. explains how the alveolar macrophage becomes activated and in turn able to stimulate the adaptive immune system and how this activation is reversed in time in the absence of continued stimulation [61].

## 3. PPARs AND THE ALVEOLAR MACROPHAGE

Macrophages differentiated from monocytes in vitro express all three isoforms of PPAR: PPAR-*α* [28], PPAR-*β*/*δ* [62], and PPAR-*γ* [28]. Activation of PPAR-*α*, but not of PPAR-*γ*, increased expression of NADPH oxidase and thereby facilitated production of reactive oxygen species [63]. Expression of some, but not all, proinflammatory molecules is decreased in macrophages isolated from PPAR-*β*/*δ* knockout mice and increased in macrophages overexpressing the receptor. However, expression of these molecules was decreased by PPAR-*β*/*δ* agonists, suggesting that it is specifically the unliganded receptor that is proinflammatory and that ligands may induce a switch between pro- and anti-inflammatory states [64]. In addition, both PPAR-*β*/*δ* and PPAR-*γ* agonists limit the ability of lipopolysaccharide to induce molecules such as nitric oxide synthase that are associated with inflammation [65].

In liver, liver-type fatty acid binding protein (L-FABP) is required for transport of both PPAR-*α* and PPAR-*γ* ligands into the nucleus [66]. Interestingly, alveolar macrophages are the only cells of the myeloid lineage to contain L-FABP [22]. Since the promoter region of the L-FABP gene contains a binding site for PPARs [66], this represents a potential signal-enhancing feed-forward mechanism.

PPAR-*γ* is known to be highly expressed in alveolar macrophages [11, 67, 68]. This is in contrast to peritoneal macrophages, where the amount is quite low in the macrophages normally resident in the peritoneum but sharply higher in activated macrophages elicited by thioglycolate [11]. The expression of PPAR-*γ* in alveolar macrophages is further upregulated by IL-4 [68]. Conversely, PPAR-*γ* is downregulated in activated peritoneal macrophages by interferon-*γ* and lipopolysaccharide [65]. Interestingly, we found that the predominant isoform in alveolar macrophages is PPAR-*γ*2, previously considered specific for adipocytes [68].

Although the amount of PPAR-*γ* in monocytes is markedly lower than in macrophages, its activation in a monocyte-like leukemia cell line has been shown to promote differentiation into cells displaying macrophage markers [27]. However, experiments with stem cells genetically lacking PPAR-*γ* have shown that this receptor is not essential for development of macrophages [69, 70].

As in many other tissues, exactly which of the many natural ligands are physiologically most important is not entirely clear. One of the highest-affinity natural ligands currently known is 15-deoxy-Δ^12,14^-prostaglandin J_2_ (15d-PGJ_2_), but levels of this molecule may be quite low in many tissues and often do not correlate with responses presumed to be mediated by PPAR-*γ* [71]. On the other hand, this ligand is plentiful in histiocytes and dendritic cells from a variety of tissues [72]. An argument for the importance of 15d-PGJ_2_ in alveolar macrophages is that lipopolysaccharide-induced synthesis of secretory type IIA phospholipase A_2_ is inhibited by arachidonic acid, a precursor of 15d-PGJ_2_ but not by its nonmetabolizable analog 5,8,11,15-tetraynoic acid [73]. Arachidonic acid is converted to 15d-PGJ_2_ by a pathway dependent on the cyclooxygenase-2 (COX-2) enzyme, and COX-2 inhibitors blocked the effect of arachidonic acid. Furthermore, the effect of arachidonic acid was mimicked by administration of either 15d-PGJ_2_ or the PPAR-*γ* ligand ciglitazone. Thus, the same effect is produced by synthetic PPAR-*γ* ligands and a metabolic precursor of 15d-PGJ_2_, suggesting that the effects observed result from binding of 15d-PGJ_2_ or a closely related compound.

It has also been shown [74] that mice lacking lysosomal acid lipase, and thus deficient in free fatty acids (including arachidonic acid), have an inflammatory phenotype in the lung that is largely eliminated by PPAR-*γ* agonists. In this case, however, the alteration is too far upstream to clearly identify the specific PPAR-*γ* ligand involved. Additionally, evidence that a given ligand plays a crucial role in one situation does not rule out involvement of different ligands in other situations.

In addition to 15d-PGJ_2_, known ligands for PPAR-*γ* include 13-hydroxyoctadecadienoic acid (13-HODE) and 15-hydroxyeicosatetraenoic acid (15-HETE), respectively produced from linoleic and arachidonic acids by 12/15-lipoxygenase. In peritoneal macrophages, the anti-inflammatory cytokine IL-4 upregulates expression of both 12/15-lipoxygenase and PPAR-*γ*, suggesting an important role for those unsaturated fatty acid derivatives in at least that specific type of macrophage [75]. 13-HODE is also found associated with oxidized LDL and is believed to play a role in regulating fatty streak macrophages [29].

Recent studies have revealed relatively large amounts of nitrated fatty acids in human blood and urine, with derivatives of oleic acid being particularly abundant [76]. These substances, which are presumably generated as a result of nitric oxide production during inflammation, have been shown to act as potent PPAR-*γ* ligands at physiological concentrations [76] and to inhibit lipopolysaccharide-induced secretion of proinflammatory cytokines by macrophages [77]. However, this latter effect was reported to reflect direct alkylation of NF-*κ*B rather than PPAR-*γ* activation. All of these natural ligands are fatty acid derivatives. The alveolus, including its resident macrophages, is constantly bathed in lipid-rich surfactant. Most of these lipids are phospholipids, but about 10% are neutral lipids including free fatty acids [78]. The essential role of free fatty acids in the production of PPAR-*γ* ligands has been demonstrated by Lian et al. [74] and by Yan et al. [79]. The former group showed that inflammation and abnormal gene expression in the lungs of lysosomal acid lipase knockout mice could be largely reversed by 9-hydroxyoctadenanoic acid or ciglitazone, while the latter group demonstrated that expression of lysosomal acid lipase specifically in macrophages had the same effect in a variety of tissues throughout the body. These results are compatible with the importance of free fatty acid release specifically within the macrophage, but it has also been shown that addition of exogenous arachidonic acid to macrophages cultured ex vivo had effects that appeared to be mediated by PPAR-*γ* [73]. Thus, PPAR-*γ* expressing cells in the alveolus are constantly bathed in precursors for the receptor's ligands. The alveolar microenvironment is immunosuppressive in the absence of specific stimulation—a conclusion supported by the finding that PPAR-*γ* binds to PPREs in resting alveolar macrophages from healthy controls but the binding is greatly reduced in those from patients with a chronic inflammatory condition such as pulmonary sarcoidosis [80].

### 3.1. Effects of PPAR-*γ* agonists on the alveolar macrophage

Early investigations of the role of PPAR-*γ* in activated peritoneal macrophages demonstrated that 15d-PGJ_2_ and rosiglitazone inhibited expression of inducible nitric oxide synthase, gelatinase B, and scavenger receptor A [11]. Similarly, in alveolar macrophages, 15d-PGJ_2_ and troglitazone inhibited the ability of lipopolysaccharide to induce synthesis of tumor necrosis factor-*α* while simultaneously upregulating expression of CD36, a scavenger receptor that mediates phagocytosis of (among other things) apoptotic neutrophils [67]; phagocytosis of apoptotic neutrophils is typical during the resolution of inflammation. In another experiment, treatment with PPAR-*γ* agonists inhibited the oxidative burst following addition of 4*β*-phorbol-12-myristate-13-acetate (PMA), expression of inducible nitric oxide synthase following treatment with lipopolysaccharide plus interferon-*γ*, and production of IL-12 following lipopolysaccharide treatment [68].

Given the role that phagocytosis of apoptotic cells plays in resolution of inflammation, it is interesting that the presence of apoptotic cells inhibits the PMA-induced oxidative burst through a mechanism that requires PPAR-*γ* [81]. Thus, PPAR-*γ* inhibits potentially destructive effects associated with inflammation, such as the release of reactive oxygen species, while facilitating the resolution of inflammation. On the other hand, however, recent work has shown that PPAR-*γ* supports noninflammatory protective effects by upregulating activity of alveolar macrophage Fc*γ* surface receptor (unpublished results) which is of particular relevance as the Fc*γ* receptor mediates phagocytosis of bacteria and other particles opsonized by attachment of antibodies belonging to the immunoglobulin G class.

Takabayashi et al. demonstrated that actin polymerization is a crucial step in the change of shape that lifts the alveolar macrophage off the epithelial cell and leads to activation [61]. Although the possible involvement of PPAR-*γ* in this process has not been directly investigated in alveolar macrophages, PPAR-*γ* agonists have been shown to inhibit actin polymerization in vascular monocytes [82].

### 3.2. PPAR-*γ* effects in other immune cells of the lung

Macrophages are not the only essential immune cells of the lung. The dendritic cell, which is also derived from monocytes and resides within the alveolar wall, takes up and processes foreign substances into antigenic fragments. These cells then migrate to the draining lymph nodes, where they present these antigens to helper T cells that promote adaptive immune responses.

Emerging data convincingly demonstrate that PPAR-*γ* agonists influence dendritic cell function. For example, exposure of dendritic cells to PPAR-*γ* ligands during differentiation in vitro results in a reduction in the ability of these cells to generate an immune response [13]. These effects include a reduction in secretion of proinflammatory cytokines and in expression of molecules essential for migration to regional draining lymph nodes where antigen presentation occurs. Dendritic cell responses to stimulation of Toll-like receptors that constitutively respond to specific molecular stimuli are likewise reduced [83]. In fact, ligands for all three PPAR isoforms reduce expression of costimulatory molecules and the ability of dendritic cells to stimulate T cells in a mixed lymphocyte reaction [84]. Furthermore, treatment of dendritic cells with PPAR-*γ* ligands during antigen-stimulated maturation (a step following differentiation) has been shown to reduce the eosinophilic response in a murine model of asthma following reinjection of the in vitro-treated cells [85]. In vivo treatment with PPAR-*γ* ligands has also been shown to inhibit migration of epidermal dendritic cells to the draining lymph nodes [86]. In one study, exposure during and after differentiation in vitro produced dendritic cells with a greatly blunted ability to release proinflammatory chemokines and, even more significantly, to activate T-lymphocytes through antigen presentation [13]. This blunted response was shown to be alleviated by administration of IL-12.

Neutrophils also play an important role in lung inflammatory responses. Although there have been numerous studies demonstrating a reduction in neutrophil-predominant inflammation following administration of PPAR-*γ* agonists [12], studies assessing direct effects on human neutrophils are limited. Most notably, we showed that PPAR-*γ* expression in neutrophils was upregulated by tumor necrosis factor-*α* or IL-4 and that incubation of resting human neutrophils with PPAR-*γ* agonists reduced chemotactic responses to IL-8 or formylmethionylleucylphenylalanine (FMLP) (unpublished results). Additionally, Imamoto et al. showed that the increased expression of neutrophil CD11b/CD18 surface integrins induced by FMLP was suppressed by pioglitazone [87].

### 3.3. Mechanisms of PPAR-*γ* action in alveolar macrophages

The molecular mechanisms through which PPAR-*γ* acts have been well characterized. What is often not explicitly appreciated is that the coactivators used by PPAR-*γ* are also used by other nuclear transcription factors, including cAMP response element-binding protein (CREB) [88, 89], activator protein-1 (AP-1) [88], basic helix-loop-helix factors [90], signal transducers and activators of transcription (STATs) [91–93], and nuclear factor-*κ*B (NF-*κ*B) [94]. Because the number of coactivator molecules is limited, PPAR-*γ* activation may restrict availability of coactivators to other nuclear transcription factors and thereby downregulate expression of genes under their control. There is also evidence that PPAR-*γ* may directly bind to these and other transcription factors, thus preventing them from binding to DNA and promoting gene transcription [87, 95–98]. Indeed, the initial report of PPAR-*γ* activation in peritoneal macrophages focused on downregulation of genes controlled by AP-1, STAT-1, and NF-*κ*B [11]. PPAR-*γ* interference with activity of NF-*κ*B also appears likely in alveolar macrophages, since PPAR-*γ* agonists inhibit the ability of lipopolysaccharide to induce synthesis of secretory type IIA phospholipase A_2_, which is promoted by NF-*κ*B [73].

Additionally, PPAR-*γ* interacts with liver X receptor-*α* (LXR-*α*). In contrast to the interactions with NF-*κ*B, however, those with LXR-*α* are synergistic [99]. Both PPAR-*γ* agonists and LXR-*α* agonists inhibit the ability of lipopolysaccharide to induce an inflammatory phenotype in cultured macrophages. However, when suboptimal concentrations of each agonist type are added simultaneously, the effects are far greater than would be seen with either agonist alone. In this study, these effects were associated with reduced activity of NF-*κ*B. Interestingly, LXR-*α* agonists increase expression and DNA binding of PPAR-*γ* [69, 99, 100], while PPAR-*γ* agonists increase LXR-*α* expression in mesangial cells [101].

## 4. PPAR-*γ* IN LUNG DISEASE

There are several lung diseases in which alveolar macrophages appear to play a crucial role in disease pathogenesis and where PPAR-*γ* agonists may prove useful as therapy. In other cases the role of the alveolar macrophage may be less clear but studies indicate that PPAR-*γ* ligands may also prove to be beneficial.

### 4.1. Pulmonary sarcoidosis

Sarcoidosis is a state of chronic granulomatous inflammation that may affect multiple organs, especially the lungs [102]. The cause of sarcoidosis remains unknown, but the pathology is characterized by greatly enhanced activation of the innate and adaptive immune systems [103, 104]. This is accompanied by increased expression of cytokines such as IL-2, IL-12, IL-18, and interferon-*γ*, with the alveolar macrophage having been demonstrated as a source for at least some of these molecules. Given the established role of PPAR-*γ* in maintaining alveolar macrophages in a quiescent state and the macrophage's role in activating other components of the immune system, examination of macrophage PPAR-*γ* levels appeared warranted. Indeed, alveolar macrophages from patients with sarcoidosis had much lower levels of PPAR-*γ* and higher levels of NF-*κ*B activity than those from healthy controls [80]. While a causal relationship between this deficiency of PPAR-*γ* and the heightened state of inflammation that characterizes sarcoidosis is plausible, the effects of PPAR-*γ* agonist administration or factors known to upregulate PPAR-*γ* expression on disease course remain to be investigated.

### 4.2. Alveolar proteinosis

Alveolar proteinosis is a condition in which excessive amounts of pulmonary surfactant, both phospholipids and proteins, accumulate in the lung airspaces [105]. Phospholipid inclusions are also prominent in alveolar macrophages [106]. Most human alveolar proteinosis (not obviously secondary to other conditions) is associated with autoantibodies to granulocyte-macrophage colony stimulating factor (GM-CSF) [107, 108]; animal models in which either GM-CSF or its receptor is genetically deleted can mimic the disease. The current treatment is removal of excess surfactant through whole-lung lavage under general anesthesia, but preliminary studies support the potential efficacy of subcutaneous GM-CSF treatment in the human disease [109, 110].

GM-CSF, which is produced by a number of cell types in the lung [111], promotes growth, differentiation, and activation of cells of the phagocytic lineage [112, 113] and has been shown to promote accumulation and proliferation of alveolar macrophages [114–116]. However, because its activities overlap those of other cytokines [117], hematopoiesis and myelopoiesis are essentially normal in GM-CSF knockout mice [118–121].

Since GM-CSF has been shown to upregulate PPAR-*γ* in cell culture [11, 75], Bonfield and colleagues examined PPAR-*γ* expression in alveolar macrophages from patients with alveolar proteinosis [122]. Not only was PPAR-*γ* mRNA and protein expression much lower than in alveolar macrophages from healthy controls, but macrophage expression of the PPAR-*γ*-dependent scavenger receptor CD36 was also lower. Furthermore, treatment with GM-CSF fully restored PPAR-*γ* to normal levels. The conclusion that GM-CSF acts at least partially through effects on macrophage PPAR-*γ* is supported by recent observations that GM-CSF also upregulates PPAR-*γ* in macrophages of the fatty streak [123].

These observations raise interesting but speculative and largely unexplored therapeutic possibilities. It could be worth considering the possibility that PPAR-*γ* agonists, including the thiazolidinediones, might prove as effective as the subcutaneous GM-CSF currently being investigated as a possible treatment.

### 4.3. Lung injury

Injury to the lung, by inhaled irritant for example, is characterized by exuberant inflammation, epithelial injury, and often the development of secondary pulmonary fibrosis. An appealing animal model of acute lung injury involves the intratracheal administration of fluorescein isothiocyanate. This insult results in a neutrophil-predominant inflammation accompanied by leakage of protein into the alveolus that is maximal at 3 to 7 days, while patchy fibrosis develops 3 to 4 weeks after exposure to the agent [124]. Using this model, we found that PPAR-*γ* expression increases in alveolar macrophages and that pretreatment with pioglitazone for 5 days prior to fluorescein isothiocyanate exposure significantly reduced inflammation and reduced the number of neutrophils in bronchoalveolar lavage fluid by 50%, but did not affect expression of proinflammatory cytokines [12]. The lack of effect on cytokine expression led us to postulate that pioglitazone was acting directly on the neutrophil to impair the ability of these cells to migrate in response to secreted chemoattractants.

In humans, alveolar macrophages isolated from patients with acute lung injury express elevated amounts of PPAR-*γ* and of PPAR-*γ* ligands such as prostaglandin D_2_ and 15-HETE [12]. Indeed, the amount of 15-HETE found in these patient's bronchoalveolar lavage fluid is more than 50 times of that seen in lavage fluid from healthy individuals. This would be expected to reduce the extent of inflammation, and hence may represent a step toward eventual resolution.

### 4.4. Other lung diseases

#### 4.4.1. Endotoxic shock

Lipopolysaccharide from gram-negative bacteria can produce severe systemic inflammation and multiorgan failure, including lung injury. The ability of PPAR-*γ* agonists to block lipopolysaccharide-induced inflammatory changes in macrophages in vitro has been well described. In an animal model, Kaplan et al. showed that this observation may have clinical relevance [125]. In this study, intraperitoneal injection of lipopolysaccharide in placebo-treated mice resulted in severe inflammatory changes in the lung, including hemorrhage, infiltration of neutrophils, and reduction of the alveolar space, that were visible within 6 hours. Increased expressions of intercellular adhesion molecule-1, vascular cellular adhesion molecule-1, and E-selectin were associated with activation of NF-*κ*B and decreased expression of PPAR-*γ*; seventy-two-hour mortality was 91%. Treatment with 15d-PGJ_2_, beginning 3 hours after lipopolysaccharide injection and continuing every 12 hours thereafter, downregulated expression of adhesion molecules, reduced neutrophil infiltration, and decreased mortality to 45%. Binding of NF-*κ*B to DNA was decreased, while expression and DNA binding of PPAR-*γ* was increased, as was expression of the protective heat shock protein 70.

Liu et al. obtained results in rats similar to those seen by Kaplan et al. in mice [126]. Specifically, rosiglitazone treatment began 30 minutes before lipopolysaccharide injection and in some cases the PPAR-*γ* antagonist GW9662 was administered 20 minutes before rosiglitazone. In the absence of rosiglitazone, lung edema and histological injury were apparent within 4 hours. These were significantly reduced by rosiglitazone, which also produced a 71% reduction of the increase in myeloperoxidase activity (a marker for the presence of neutrophils) and an 84% reduction of the increase in malondialdehyde levels. This was accompanied by a marked decrease in inducible NO synthase mRNA and protein. All these rosiglitazone effects were blocked by the PPAR-*γ* antagonist GW9662.

Although these are animal studies and do not directly demonstrate involvement of either PPAR-*γ* or alveolar macrophages in the effects observed, they suggest that early treatment with PPAR-*γ* agonists could ameliorate the effects of endotoxemia, at least in the lung and probably elsewhere.

#### 4.4.2. Asthma

The alveolar macrophage has been described as “the forgotten cell in asthma” [127]. Asthma is an exaggerated response of the lung's adaptive immune system to specific inhaled antigens—a response that the alveolar macrophage downregulates in most circumstances. It is thus not surprising that depletion of alveolar macrophages led to an enhanced response to challenge with an antigen to which mice had previously been sensitized [128]. As discussed by Peters-Golden, alveolar macrophages exert a variety of effects that could lead to suppression of exaggerated asthmatic responses [127] and many of these are precisely the effects that are elicited and maintained by PPAR-*γ*. Importantly, allergen challenge in asthmatic patients has been found to downregulate PPAR-*γ* levels in alveolar macrophages [129]. Otherwise, however, the contribution of PPAR-*γ* to alveolar macrophage responses in asthma has not been explicitly investigated.

## 5. CONCLUSIONS

Among the many often-overlooked roles of PPAR-*γ* is its central position in regulating the lung's response to pathogens and other noxious elements drawn in with inspired air. The lung must be able to respond effectively, yet to control the inflammatory response generated in response to foreign agents within the alveolar space. The alveolar macrophage is pivotal in this respect. The ability of these cells to engulf unwanted particles represents the first line of defense, yet when not fully activated (e.g., by interactions with its Toll-likereceptors) serves to strongly dampen responses by the lung's adaptive immune system. However, when these cells have been sufficiently activated by danger signals within the alveolus, alveolar macrophages release molecules that attract and activate other elements of the innate and adaptive immune systems. Finally, the macrophage's ability to scavenge apoptotic neutrophils is essential for resolution of inflammation once the need has passed.

**Figure 1:**
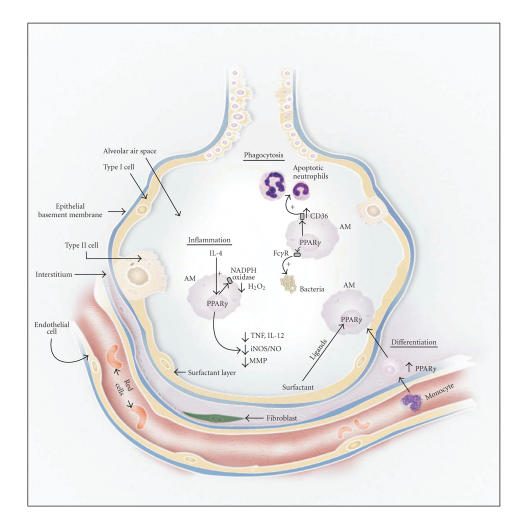
*The alveolar macrophage: role in immunity and effects of PPAR-*γ**. PPAR-*γ* promotes differentiation of monocytes into AMs, then mediates effects that suppress AMs' proinflammatory activities while upregulating phagocytosis through both CD36 and Fc*γ* surface receptors. Interaction with foreign substances causes the AM to secrete a variety of inflammatory molecules such as TNF-*α*, IL-12, H_2_O_2_, nitric oxide, and MMPs. This inflammatory response is suppressed, however, by the production of substances such as IL-4 that reinforce PPAR-*γ* activity. Alveolar surfactant also contains lipids that can stimulate PPAR-*γ*, resulting in suppression of AM inflammatory activity. PPAR-*γ* activation also enhances AM phagocytosis of bacteria and apoptotic neutrophils present in the closing stages of inflammation. Thus, inflammation becomes self-limiting. Abbreviations: AM = alveolar macrophage; Fc*γ*R = surface receptor recognizing the Fc portion of immunoglobulin G; iNOS = inducible nitric oxide synthase; MMP = matrix metalloproteinase.

The central role of PPAR-*γ* in regulating the activational state of alveolar macrophages is becoming increasingly clear (see Figure 1). Many studies have now shown that macrophage activation is inhibited by PPAR-*γ* and/or PPAR-*γ* agonists. This is typically associated with decreases in NF-*κ*B activity, with one likely mechanism being unavailability of that transcription factor's obligate coactivators because they are being used by PPAR-*γ* instead. Conversely, activation of alveolar macrophages is associated with low levels of PPAR-*γ* and high levels of NF-*κ*B activity.

Many aspects of alveolar macrophage function and the role of PPAR-*γ* in regulating these functions remain unclear. Especially uncertain are how these responses contribute to pathological conditions such as asthma and acute lung injury. All of these aspects deserve further investigation, with special attention to the possibility that PPAR-*γ* agonists may prove therapeutically useful in a variety of lung diseases in which they have not previously been considered.

## LIST OF ABBREVIATIONS

13-HODE: 13-hydroxyoctadecadienoic acid
15d-PGJ_2_: 15-deoxy-Δ^12,14^-prostaglandin J_2_
15-HETE: 15-hydroxyeicosatetraenoic acid
AP-1: Activator protein-1
COX-2: Cyclooxygenase-2
CREB: cAMP response element-binding protein
FMLP: Formylmethiolylleucylphenylalanine
GM-CSF: Granulocyte-macrophage colony stimulating factor
IL-: Interleukin-
L-FABP: Liver-type fatty acid binding protein
LDL: Low-density lipoprotein
LXR: Liver X receptor
MMP: Matrix metalloproteinase
NF-*κ*B: Nuclear factor-*κ*B:
PMA: 4*β*-phorbol-12-myristate-13-acetate
PPAR: Peroxisome proliferator-activated receptor
STAT: Signal transducer and activator of transcription
TGF: Transforming growth factor
